# The Hippo pathway acts downstream of the Hedgehog signaling to regulate follicle stem cell maintenance in the *Drosophila* ovary

**DOI:** 10.1038/s41598-017-04052-6

**Published:** 2017-06-30

**Authors:** Ta-Hsing Hsu, Chia-Yu Yang, Tsung-Han Yeh, Yi-Chia Huang, Tsu-Wei Wang, Jenn-Yah Yu

**Affiliations:** 1 Department of Life Sciences and Institute of Genome Sciences, National Yang-Ming University, Taipei, 112 Taiwan; 2 Department of Life Science, National Taiwan Normal University, Taipei, 116 Taiwan; 3 Brain Research Center, National Yang-Ming University, Taipei, 112 Taiwan

## Abstract

The Hippo pathway is conserved and plays important roles in organ size control. The core components of the Hippo pathway are two kinases Hippo (Hpo), Warts (Wts), and a transcription-co-activator Yorkie (Yki). Yki activity is regulated by phosphorylation, which affects its nuclear localization and stability. To determine the role of the Hippo pathway in stem cells, we examine follicle stem cells (FSCs) in the *Drosophila* ovary. Yki is detected in the nucleus of FSCs. Knockdown of *yki* in the follicle cell lineage leads to a disruption of the follicular epithelium. Mitotic clones of FSCs mutant for *hpo* or *wts* are maintained in the niche and tend to replace the other FSCs, and FSCs mutant for *yki* are rapidly lost, demonstrating that the Hippo pathway is both required and sufficient for FSC maintenance. Using genetic interaction analyses, we demonstrate that the Hedgehog pathway acts upstream of the Hippo pathway in regulating FSC maintenance. The nuclear localization of Yki is enhanced when the Hedgehog signaling is activated. Furthermore, a constitutively active but not a wild-type Yki promotes FSC maintenance as activation of the Hedgehog signaling does, suggesting that the Hedgehog pathway regulates Yki through a post-translational mechanism in maintaining FSCs.

## Introduction

Stem cells undergo self-renewal to produce daughter cells with identical properties of the mother cell and daughter cells that differentiate into different types of cells. During developmental and adult stages, stem cells play critical roles to sustain tissue growth and homeostasis by expanding cell numbers and replacing aged or injured cells. Thus, it is important to understand molecular mechanisms underlying stem cell maintenance. Extrinsic signals and adherence molecules provided by the surrounding microenvironment, known as the niche, are essential for stem cells maintenance^1, 2^. Intrinsic signaling networks are also necessary for regulating stem cell fate and differentiation^3^.

To study signaling pathways regulating stem cells, the *Drosophila* ovary is an excellent model given its simple structure and convenient genetic tools^4^. The *Drosophila* ovary is comprised by fifteen to twenty tubular structures called ovarioles^5^. The most anterior part of the ovariole is a structure called the germarium, which contains germline stem cells (GSCs) and follicle stem cells (FSCs)^6^. Two to three GSCs are located at the anterior tip of the germarium, where they continuously divide to produce germline cysts^7^. Once the germline cyst passes through the junction between regions 2a and 2b of the germarium, it is wrapped by the follicular epithelium derived from FSCs. There are two FSCs in each germarium^8^. FSCs produce follicle cell precursors which differentiate into three types of cells: polar cells, stalk cells, and main-body follicle cells. Polar cells are specified early during oogenesis and play important roles in axis determination of the oocyte. Stalks cells separate adjacent egg chambers. Main-body follicle cells encapsulate the germline cyst to form the egg chamber. From cell division of GSCs to a mature egg, the process of oogenesis can be divided into fourteen stages based on the size and cell cycle status of germline and follicle cells^9^. Main-body follicle cells undergo eight to nine rounds of cell divisions to maintain a mono-layered epithelium surrounding the egg chamber before they enter endocycle at stage 6. These follicle cells are important for yolk production and eggshell formation in the following steps of oogenesis^10, 11^.

The FSC in the germarium is a well-characterized epithelial stem cell^12^. Many adhesion molecules and signaling pathways have been shown to contribute to FSC maintenance. FSCs mutant for *shotgun* (*shg*), which encodes *Drosophila* E-Cadherin (DE-Cad), are rapidly lost from the germarium, demonstrating that the adherence to nearby cells is required for FSC maintenance^13^. Escort cells immediately adjacent to FSCs serve as dynamic niche cells by providing ligands of Hedgehog pathway^14^. In addition to escort cells, cap cells in the anterior region of the germarium also secrete BMP and Wingless ligands to maintain FSCs^15, 16^. Interestingly, a recent study suggests that FSC maintenance is controlled by coordinative activity of both Hedgehog and JAK-STAT pathways^17^. Hedgehog (Hh) is released from the terminal filament and cap cells^18^. For the Hedgehog pathway, a transmembrane protein Patched (Ptc) constitutively represses the signaling activity by inhibiting Smoothened (Smo), a seven-transmembrane protein belonging to the G protein-coupled receptor (GPCR) superfamily^19^. The interaction of Hh and Ptc results in Smo activation, which acts through intracellular signaling complexes to convert the transcription factor Cubitus interruptus (Ci) from a truncated repressor to a full-length activator^20^. FSCs mutant for *ptc* are strongly maintained in the niche; and FSCs mutant for *smo* or *ci* are rapidly lost^21^. The JAK-STAT ligand Unpaired (Upd) is secreted from polar cells. FSCs mutant for genes encoding for *Drosophila* Janus Kinase *Hopscotch* (*Hop*) or Stat *Stat92E*, are lost from the niche^17^. Together, these data demonstrate that multiple signaling pathways contribute to FSC maintenance. Recently, a key component of the Hippo pathway Yorkie (Yki) has also been suggested to regulate FSC maintenance downstream of the co-activator of Notch signaling Mastermind (Mam) and the Hedgehog pathway^22^. However, the molecular mechanism underlying the interaction between the Hippo and Hedgehog pathways remains unclear.

The Hippo pathway is conserved from *Drosophila* to human and important for organ size control. Its core components include Hippo (Hpo), Warts (Wts), Salvador (Sav), Mob as a tumor suppressor (Mats), and Yorkie (Yki). Hpo is an Ste20-like kinase that forms a complex with the adaptor protein Sav and phosphorylates Wts, a nuclear Dbf2-related (NDR) family kinase^23–26^. In association with the adapter protein Mats^27^, Wts phosphorylates Yki, a transcriptional coactivator^28^. Phosphorylated Yki is retained in the cytosol and degraded^29, 30^. When Yki is not phosphorylated, it is imported to the nucleus and interacts with transcription factors such as Scalloped (Sd) to induce target genes such as *cyclin E*, *Myc*, *Diap*, *expanded* (*ex*), or *bantam* to promote cell proliferation and survival^31^. Interestingly, gene expression profiles do not overlap much among studies with different tissues or cells in *Drosophila* and mammals^32–36^, suggesting that Yki and its homologues YAP/TAZ in mammals may control gene expression in a tissue-specific manner. Thus, we should be cautious to take the expression of Yki target genes as indication of the transcriptional activity of Yki in specific cell types or tissues. Since the Hippo pathway is critical in growth control, various signals act upstream of the Hippo pathway, including cell polarity and cell junctions, cell adhesion, cell-cell contact and mechanical cues, cytoskeletons, G protein-coupled receptor signaling and cell metabolic status^37^. Moreover, the Hippo pathway interacts with Wnt, BMP, Notch, and Hedgehog pathways in various tissues and organisms^38^. Thus, it is important to understand how the Hippo pathway integrates such complicated signaling networks to modulate cellular functions.

We have previously discovered roles of the Hippo pathway in cell fate determination and migration of the follicle cell lineage^39, 40^. In this study, we showed that the Hippo pathway is both required and sufficient for FSC maintenance. We further used genetic interactions to demonstrate that the Hippo pathway acts downstream of the Hedgehog signaling. While *ptc* mutant FSCs were maintained, *ptc* and *yki* double-mutant FSCs were lost quickly as that of *yki* mutant FSCs. Reduction of *wts* partially rescued the maintenance defect of *smo* mutant FSCs. Both the level and nuclear distribution of Yki were increased in *ptc* mutant clones. Importantly, over-expression of a constitutively active but not wild-type *yki* promoted FSC maintenance as over-activation of the Hedgehog pathway did, suggesting that the Hedgehog pathway regulates Yki activity through a post-translational mechanism. Together, our data demonstrate that the Hippo pathway acts downstream of the Hedgehog signaling in regulating FSC maintenance.

## Results

### Yki is detected in the follicle cell lineage

**Figure 1:**
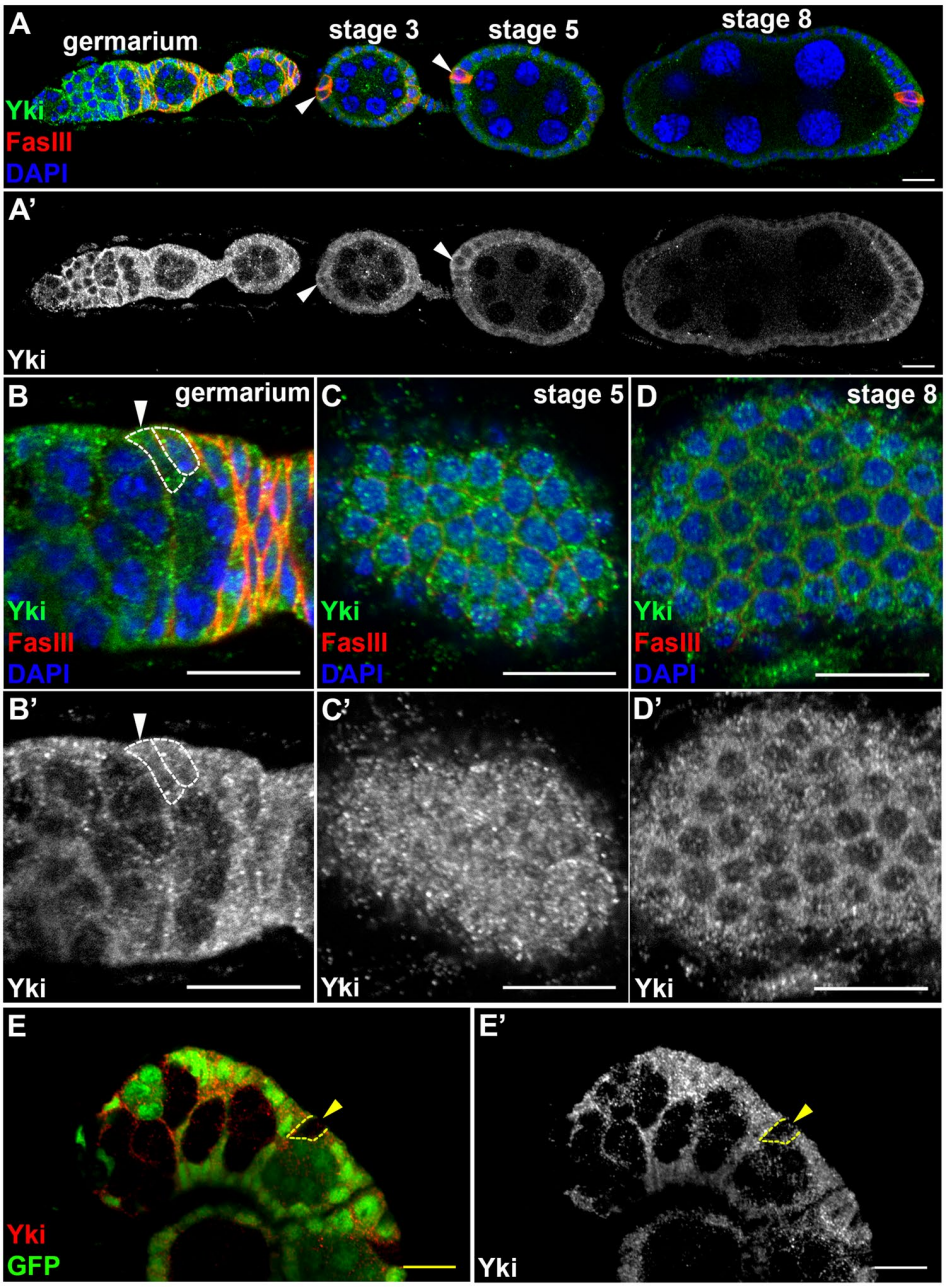
The level and distribution of Yki in the follicle cell lineage. Ovaries from *w* ^*1118*^ were stained with anti-Yki, anti-FasIII or anti-GFP. Nuclear DNA was stained with DAPI. Samples were oriented as anterior to the left. (**A**) Immunostaining pattern of Yki in an ovariole with the germarium and egg chambers at stages 2, 3, 5 and 8. Yki was detected in the follicle lineage. The level of Yki was high in FSCs and follicle cell precursors, and decreased gradually. In polar cells (white arrowheads) and follicle cells at stage 8, Yki was excluded from the nucleus. (**B**) A high level of Yki was observed in FSCs (white arrowheads). Yki was detected at similar level in both the cytoplasm and nucleus. (**C**) A high level of Yki was detected in main-body follicle cells prior to stage 5. The levels of Yki were similar in the cytoplasm and nucleus. (**D**) At stage 8, the Yki level was decreased and excluded from the nucleus. (**E**) No Yki signal was detected in GFP-negative *yki* mutant clones (yellow arrowheads) six days after clone induction. Scale bar length is 10 μm.

To investigate roles of the Hippo pathway during oogenesis, we first examined the expression pattern of Yki by generating a polyclonal antibody against Yki (anti-Yki). Yki was detected in the follicle cell lineage but not germline cells (Fig. 1A). To distinguish different follicle cell types, we stained ovaries with anti-Fasciclin III (FasIII), which labels follicle cell precursors in the germarium and polar cells in the developing egg chamber (Fig. 1A)^41^. FSCs were identified by their localization between 2a and 2b regions of the germarium and a low level of FasIII (Fig. 1A,B). The immunofluorescent staining pattern of our anti-Yki is consistent with that of the anti-Yki from Dr. Kenneth Irvine’s laboratory^29, 39^. Since Yki is a transcriptional co-activator, its nuclear localization is critical for its function^29, 42^. In the follicle cell lineage, Yki was detected in the nucleus of FSCs, follicle cell precursors, and follicle cells prior to stage 5 when they were in mitotic cell cycle (Fig. 1A–C). Importantly, Yki was not detected in the nucleus of polar cells, which withdrew from cell cycle after stage 2 (Fig. 1A)^39^. Yki was not detected in the nucleus of follicle cells after stage 5 when they entered endocycle (Fig. 1D). No immunofluorescent signal was detected in *yki* mutant clones, demonstrating the specificity of our anti-Yki (Fig. 1E). Yki activity has been examined previously by using reporters such as *Diap-LacZ* and *ex-LacZ* in the intestine and discs, where cells continuously undergo proliferation^28, 43–45^. Unexpectedly, both *Diap-LacZ* and *ex-LacZ* were expressed in polar cells and follicle cells withdrawn from mitosis at stage 7 (Supplementary Fig. S1). These cells expressing *ex-LacZ* and *diap-LacZ* did not show nuclear localization of Yki. While functional analyses have demonstrated that Yki functions to inhibit polar cell fate and promote mitosis in follicle cells^39, 46^, it is likely that Yki does not target *ex* or *Diap* in the follicle cell lineage. To further examine whether the Hippo pathway regulates the expression of *Diap-LacZ* and *ex-LacZ*, we generated *yki* or *hpo* mutant clones in the background of *Diap-LacZ* as well as *wts* mutant clones in the background of *ex-LacZ*. The intensities of β-GAL in *yki* mutant follicle cell precursors and follicle cells at stage 5 were not significantly reduced comparing with the neighboring GFP-positive control cells (Fig. S2A,B). Consistently with previous findings^46, 47^, *Diap-LacZ* was strongly increased in *hpo* mutant posterior follicle cells and *ex-LacZ* was strongly increased in *wts* mutant posterior follicle cells after stage 6 (Fig. S2D,F). However, the intensity of β-GAL in *hpo* or *wts* mutant follicle cell precursors was similar to that of the neighboring GFP-positive control cells (Fig. S2C,E). This result support that the Hippo pathway does not regulate *ex* or *Diap* in the follicle cell lineage during early oogenesis. Therefore, we used the level and distribution of Yki immunofluorescent staining instead of those Yki activity reporters in our following experiments.

### Yki is required for maintaining the follicle cell lineage

**Figure 2:**
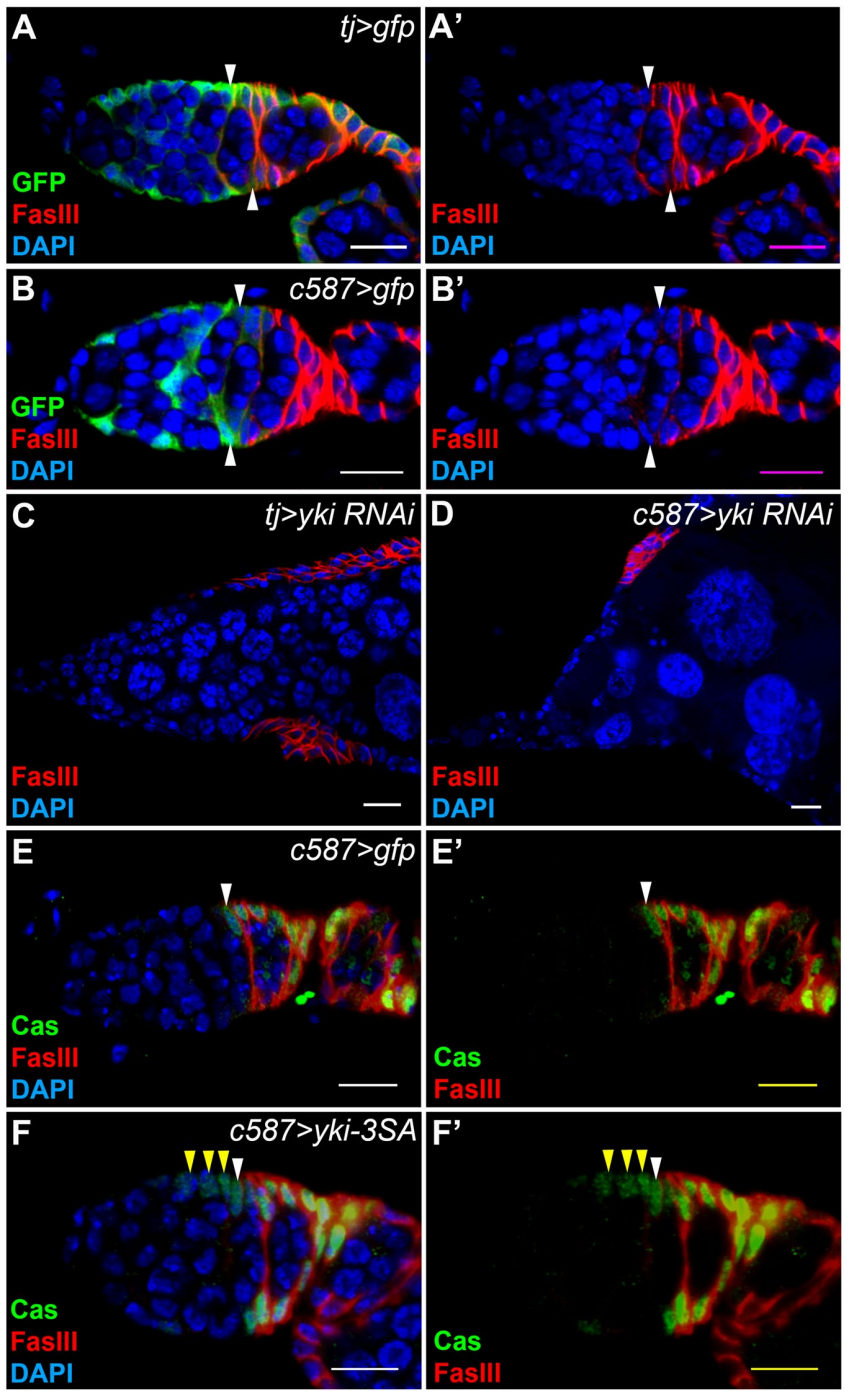
Yki is crucial for the follicle cell lineage. The flies were grown at 29 °C for six days. Ovaries were dissected and stained with anti-FasIII, anti-GFP (**A**,**B**), anti-Cas (**E**,**F**) and DAPI. Samples were oriented as anterior to the left. (**A**) *tj-GAL4* was used to drive *gfp* expression. GFP was detected in escort cells, FSCs (white arrowheads), follicle cell precursors and main-body follicle cells. (**B**) *c587-GAL4* was used to drive *gfp* expression. GFP was detected in escort cells and FSCs (white arrowheads), but not in follicle precursors or follicle cells. (**C**,**D**) When *yki-RNAi* was driven by *tj-GAL4* or *c587-GAL4*, the entire follicular epithelium was disrupted. Ectopic FasIII-positive cells were observed. (**E**) FSCs (white arrowheads) and follicle cell precursors were Cas-positive in the germarium of *c587-GAL4*. (**F**) *yki-3SA* was driven by *c587-GAL4*. In the germarium, ectopic Cas-positive cells anterior to the FSC were observed (yellow arrowheads). Scale bar length is 10 μm.

Since Yki was localized in the nuclei of FSCs, we further examined the function of Yki by using UAS-GAL4 system to knock down or over-express *yki*. By crossing with a *UAS-gfp* line, we showed that *traffic jam* (*tj*)-*GAL4* was expressed in most cells of the follicle cell lineage including FSCs and *c587-GAL4* was expressed abundantly in FSCs and escort cells (Fig. 2A,B). Knockdown of *yki* with either *tj-GAL4* or *c587-GAL4* severely disrupted the follicular epithelium (Fig. 2C,D), which might be resulted from the loss of FSCs. Most remaining follicle cells were positive for FasIII (Fig. 2C,D). Although FasIII is detected in both follicle cell precursors and polar cells, our previous study clearly demonstrates that loss of Yki function promotes polar cells differentiation^39^. Thus it is likely that these FasIII-positive cells resulted from *yki* knockdown are polar cells (Fig. 2C,D). Nuclear localization and activity of Yki is regulated by Ser/Thr phosphorylation. Ser residues at positions 111, 168, and 250 of Yki are phosphorylated by Wts and the phosphorylation prevents its nuclear localization. Since the Ser 111, 168, 250 to Ala mutations of Yki promote its nuclear import, we over-expressed *yki-S111A, S168A, S250A* (*yki-3SA*) instead of the wild-type *yki*. Previous studies have shown that Castor (Cas) is detected in FSCs and follicle cell precursors^48^ (Fig. 2E). Over-expression of *yki-3SA* with *c587-GAL4* increased Cas-positive cells anterior to the region 2a/2b junction (Fig. 2F), suggesting that constitutive activation of Yki might result in ectopic FSCs. Together, our data suggested that Yki plays an important role in the follicle cell lineage, possibly in FSCs.

### The Hippo pathway regulates follicle stem cell maintenance

**Figure 3:**
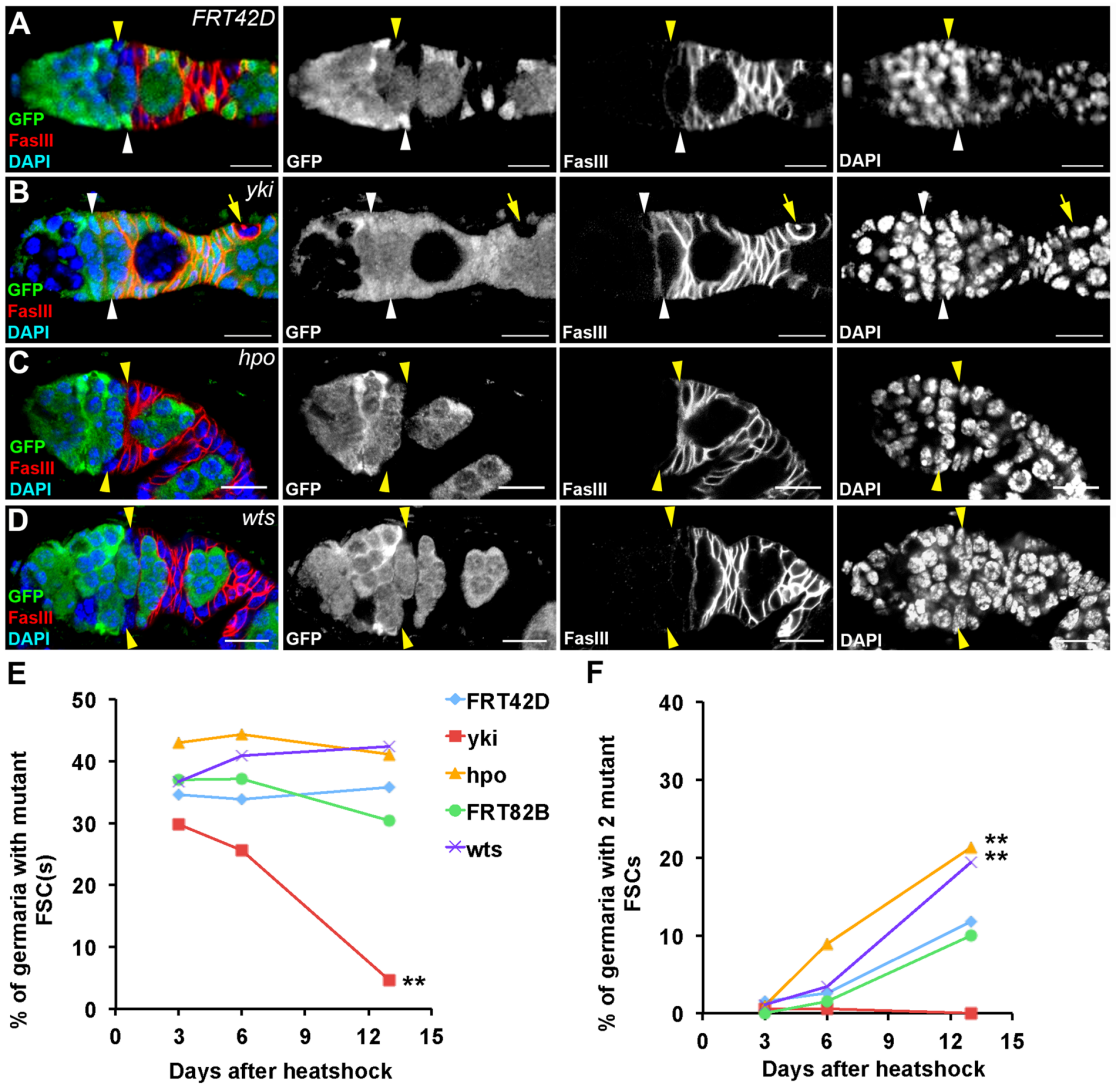
The Hippo pathway regulates FSC maintenance. Ovaries were dissected thirteen days after clone induction and stained with anti-GFP, anti-FasIII, and DAPI. Mitotic clones were GFP-negative. (**A**) A germarium with one GFP-negative (yellow arrowheads) and one GFP-positive (white arrowheads) FSCs in *FRT42D* control. (**B**) A germarium with two GFP-positive FSCs (white arrowheads). FasIII-positive *yki* mutant cells were observed in a stage 2 egg chamber (yellow arrows). (**C**) A germarium with two GFP-negative *hpo* mutant FSCs (yellow arrowheads). (**D**) A germarium with two GFP-negative *wts* mutant FSCs (yellow arrowheads). (**E**,**F**) Germaria were categorized based on the numbers of GFP-negative FSCs in a germarium three, six, and thirteen days after clone induction. Pearson’s chi-squared test was used for statistic analysis. *n* ≥ 180 for each genotype/time point. **p < 0.01. (**E**) The percentages of germaria with one or two GFP-negative FSCs were shown. The percentages of germaria with one or two *FRT42D*, *FRT82B*, *hpo*, or *wts* mutant FSCs were maintained over time. The percentages of germaria with one or two *yki* mutant FSCs were decreased drastically. (**F**) The percentages of germaria with two GFP-negative FSCs were shown. The percentages of germaria with two *FRT42D* or *FRT82B* GFP-negative FSCs were increased slowly. Few germarium with two *yki* mutant FSCs was observed six or thirteen days after clone induction. The percentage of germaria with two *hpo* or *wts* mutant FSCs were increased dramatically. Scale bar length is 10 μm (**A**–**D**).

We next examined the maintenance of FSCs mutant for *yki*, *hpo* or *wts*. Mitotic clones generated by the Flipase/FRT system were identified by not expressing GFP (Fig. 3A–D). *FRT42D* and *FRT82B* clones were generated as controls. After clone induction, ovaries were dissected at different time points and the percentage of germaria containing mutant FSC clones was calculated (Fig. 3E,F). In *FRT42D* and *FRT82B* groups, most GFP-negative FSCs were maintained in the germarium thirteen days after clone induction (Fig. 3A,E, Fig. S3A). The percentage of germaria containing two GFP-negative *FRT42D* or *FRT82B* FSCs increased slowly after clone induction, suggesting that some GFP-positive FSCs have been replaced by their neighboring GFP-negative FSCs (Fig. 3F). *yki* mutant FSCs were rapidly lost and differentiated into FasIII-positive polar cells (Fig. 3B,E)^39^. Most *hpo* or *wts* mutant clones were maintained in the germarium (Fig. 3C–E). Importantly, more germaria contained two *hpo* or *wts* mutant FSCs than that of the *FRT42D* and *FRT82B* control groups thirteen days after clone induction, suggesting that *hpo* or *wts* mutant FSCs tend to stay in the niche and replace the neighboring GFP-positive control FSC (Fig. 3F). Consistently, *sav* mutant FSCs tended to replace the GFP-positive neighboring control FSC (Fig. S3B,E); *sd* mutant FSCs were rapidly lost from the germarium (Fig. S3D, F). These data are consistent with a previous study and demonstrate that core components of the Hippo pathway are important for regulating FSC maintenance^22^.

### Confirmation of the interaction between the Hippo and Hedgehog pathways

**Figure 4:**
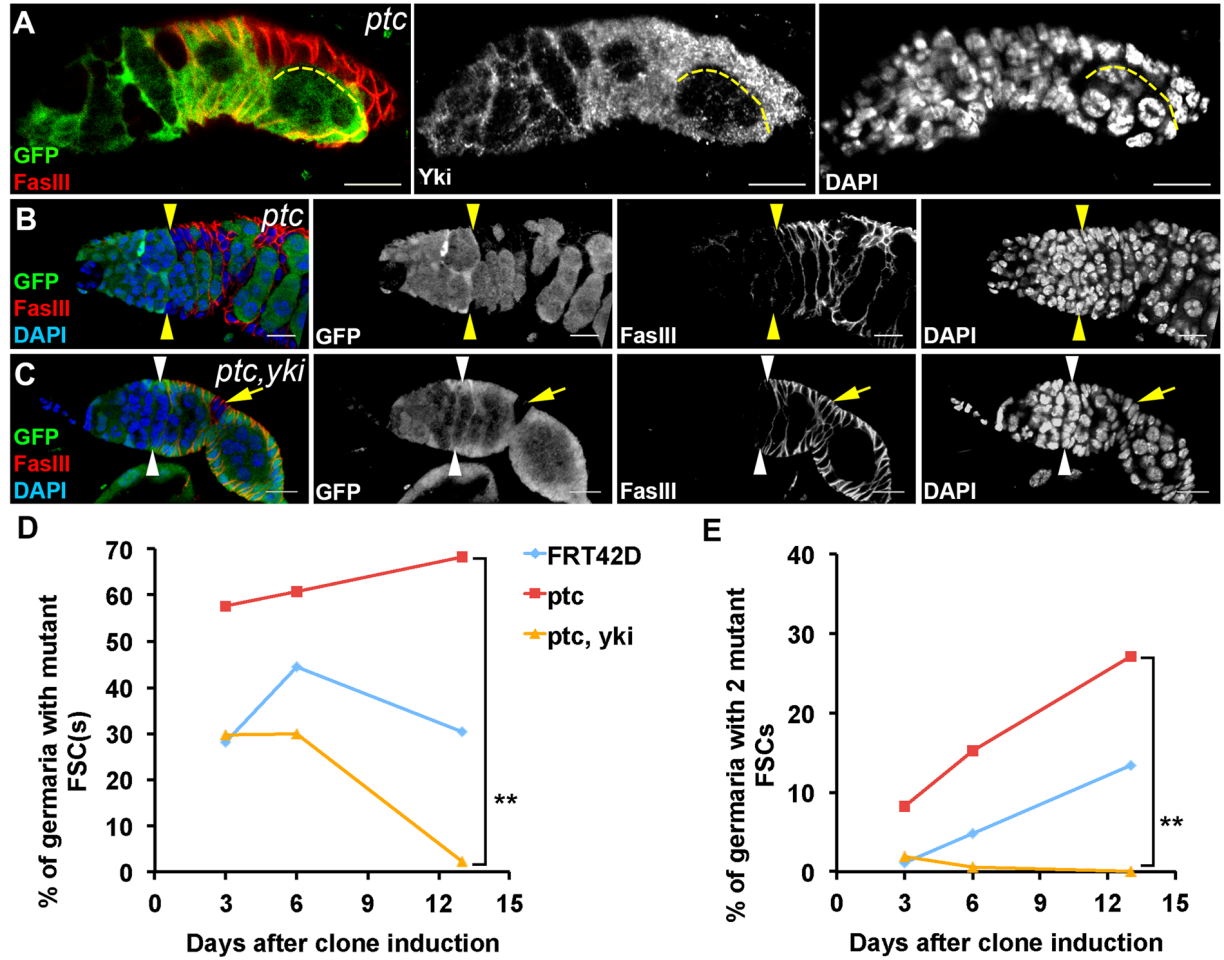
Yki acts downstream of the Hedgehog pathway in promoting FSC maintenance. Ovaries were dissected six days (**A**) or thirteen days (**B**,**C**) after clone induction and stained with anti-GFP, anti-FasIII, anti-Yki (**A**,**B**), and DAPI. Mitotic clones were GFP-negative. (**A**) The intensity of Yki immunofluorescent staining was increased in *ptc* mutant follicle cells in the germarium. (**B**) A germarium with two GFP-negative *ptc* mutant FSCs (yellow arrowheads) were shown. (**C**) A germarium with two GFP-positive FSCs (white arrowheads). *ptc*, *yki* double mutant cells left the germarium and were FasIII-positive (yellow arrows). (**D**) The percentages of germaria with one or two *ptc*, *yki* double mutant FSCs were decreased drastically over time. (**E**) The percentages of germaria with two GFP-negative *FRT42D* control FSCs were increased slowly over time. No germarium with two *ptc*, *yki* double mutant FSCs was observed thirteen days after clone induction. The percentage of germaria with two *ptc* mutant FSCs were increased dramatically. Pearson’s chi-squared test was used for statistic analysis. *n* ≥ 180 for each genotype/time point. Scale bar length is 10 μm.

Similar to the Hippo pathway, it has been shown that the Hedgehog pathway is required and sufficient for FSC maintenance^21^, so we tested whether the Hippo and Hedgehog pathways may interact with each other in the follicle cell lineage. We have previously shown that mutation of *wts* did not affect the expression of *ptc-lacZ*, a reporter for the Hedgehog signaling activity^39^, suggesting that the Hippo pathway does not act upstream of the Hedgehog pathway. It has been demonstrated that the Hedgehog pathway acts upstream of the Hippo pathway in regulating FSC maintenance based on the expression of *Diap-LacZ* ^22^. Since the expression of *Diap-LacZ* does not reflect the Hippo pathway activity during early oogenesis (Fig. S2), we examined the level of Yki in *patched* (*ptc*) mutant cells. Ptc is a key negative regulator of the Hedgehog pathway. The Yki level was significantly increased in *ptc* mutant cells in follicle cell precursors (Fig. 4A). This result confirmed that the Hippo pathway acts downstream of the Hedgehog signaling in the follicle cell lineage.

We further examined the genetic interaction between the Hippo and Hedgehog pathway demonstrated previously by Kalderon’s laboratory^22^. *ptc* mutation leads to over-activation of the Hedgehog pathway and strongly promotes FSC maintenance^21^. If the Hippo pathway acts downstream of the Hedgehog pathway, *ptc yki*-double mutant FSCs should be lost from the germarium as *yki* mutant FSCs. *FRT42D* clones were generated as controls. Consistent with previous results^21^, *ptc* mutant FSCs were maintained in the germarium and more germaria contained two *ptc* mutant FSCs than that of the *FRT42D* control thirteen days after clone induction (Fig. 4B,D and E), confirming that *ptc* mutant FSCs tend to replace the GFP-positive neighboring control FSCs. Similar to that of *yki* mutant FSCs, *ptc yki*-double mutant FSCs were rapidly lost (Fig. 4C–E), supporting our hypothesis that the Hippo pathway acts downstream of the Hedgehog pathway in regulating FSC maintenance.

**Figure 5:**
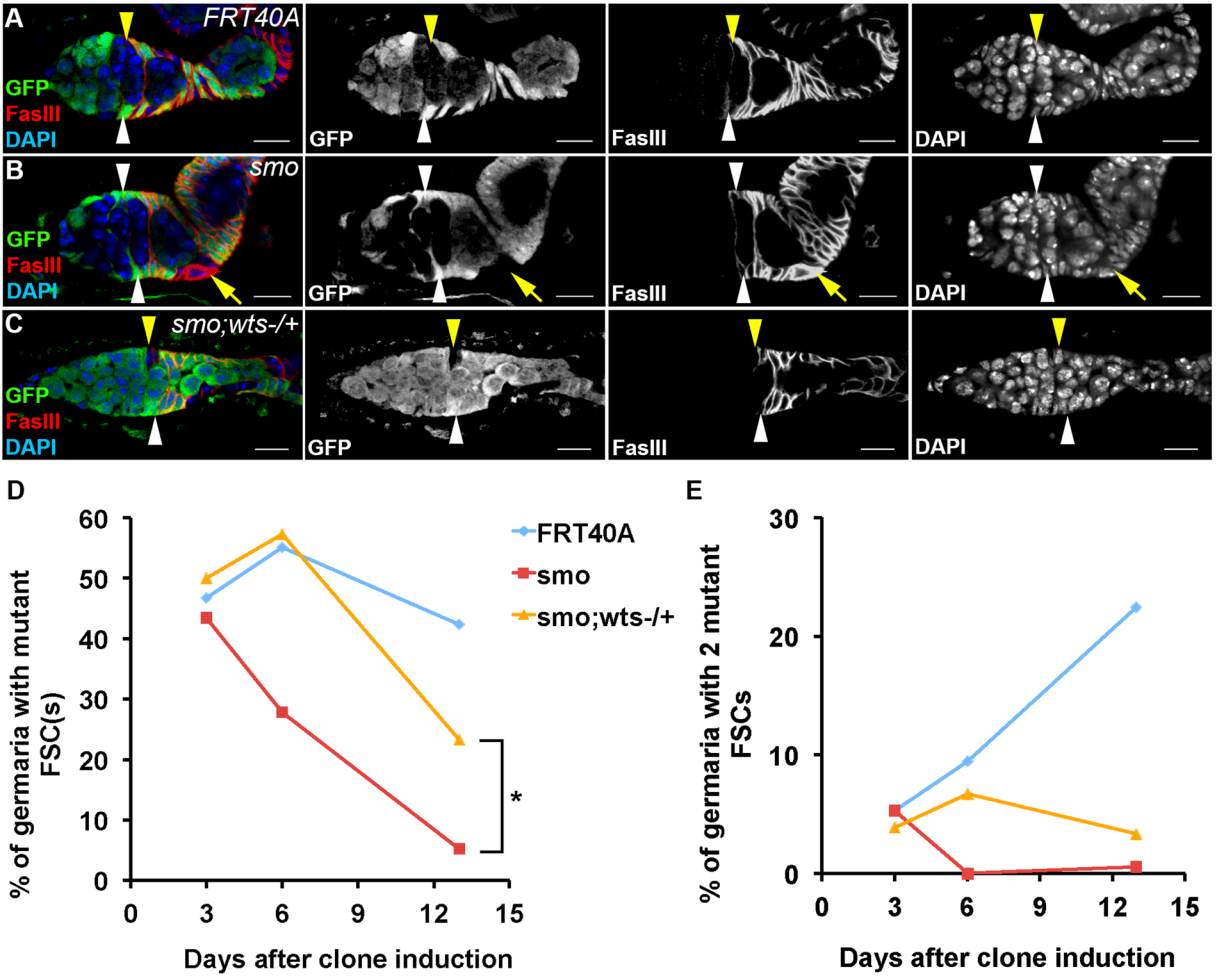
Reduction of *wts* promotes maintenance of *smo* mutant FSCs. Ovaries were dissected thirteen days after clone induction and stained with anti-GFP, anti-FasIII, and DAPI. Mitotic clones were GFP-negative. (**A**) A germarium with one GFP-negative (yellow arrowheads) and one GFP-positive (white arrowheads) *FRT40A* control FSC were shown. (**B**) A germarium with two GFP-positive FSCs (white arrowheads) were shown. *smo* mutant cells left the niche (yellow arrows). (**C**) A germarium with one *smo* mutant GFP-negative FSC (yellow arrowheads) and one GFP-positive FSC (white arrowheads) in a *wts* heterozygous background were shown. (**D**) The percentage of germaria with one or two *smo* mutant FSCs was decreased drastically. In a *wts* heterozygous mutant background, however, the percentage of germaria containing one or two *smo* mutant FSCs was increased. (**E**) The percentages of germaria with two GFP-negative *FRT40A* FSCs were increased slowly. Few germarium with two *smo* mutant FSCs was observed. In the *wts* heterozygous mutant background, some of germaria with two *smo* mutant FSCs were observed. Pearson’s chi-squared test was used for statistic analysis. *n* ≥ 180 for each genotype/time point. Scale bar length is 10 μm.

We further tested whether reduction of *wts* might promote maintenance of FSCs mutant for *smo*, a positive regulator of the Hedgehog pathway. *FRT40A* clones were used as a control. Comparing with *FRT40A* control FSCs, *smo* mutant FSCs were rapidly lost (Fig. 5A,B and D). In a *wts* heterozygous background, the percentage of germaria containing *smo* mutant FSCs were increased at thirteen days after clone induction (Fig. 5C,D). However, the percentage of germaria containing two *smo* mutant FSCs were not rescued by reduction of *wts* (Fig. 5E). Thus, reduction of *wts* partially rescued the FSC maintenance defect of *smo* mutation. Our data provided further evidence that the Hippo pathway acts downstream of the Hedgehog pathway in regulating FSC maintenance.

### The Hedgehog pathway regulates the Hippo pathway through a post-transcriptional mechanism

**Figure 6:**
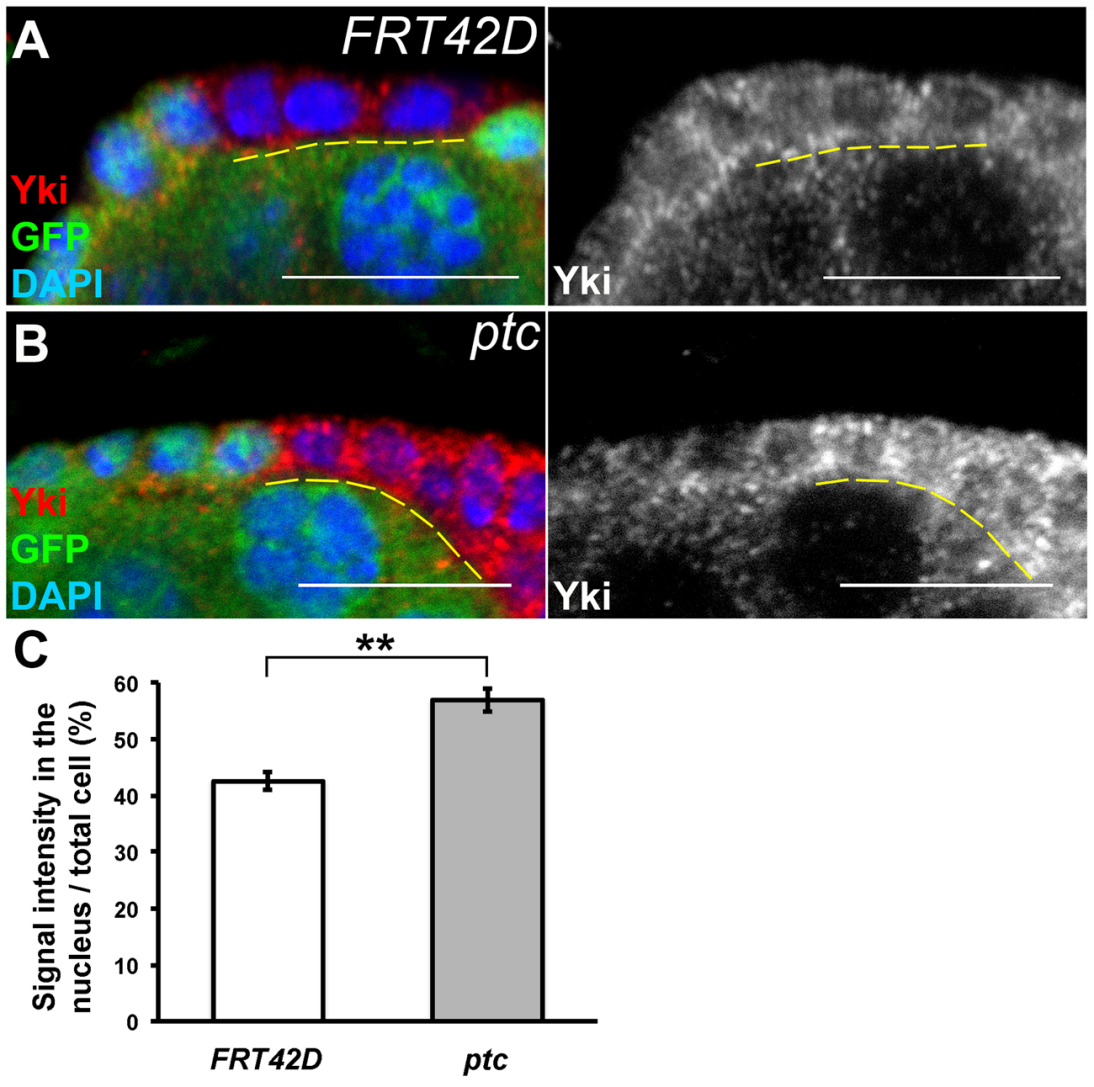
The Hedgehog pathway regulates nuclear distribution of Yki. Ovaries were dissected six days after clone induction and stained with anti-GFP, anti-Yki, and DAPI. Stage 3 to 4 egg chambers were selected. Mitotic clones were GFP-negative. (**A**) In *FRT42D* control clones, Yki was detected in both nucleus and cytoplasm. (**B**) In *ptc* mutant, the levels of Yki in the whole cell as well as in the nucleus were increased. (**C**) Quantification of the intensity of Yki immunofluorescent staining and the ratio of the intensity in the nucleus to that of the whole cell. The ratio was significantly higher in *ptc* mutant cells than that of FRT42D control cells. The bar graph is shown as Mean ± SEM. Student’s t-test is used for statistic analysis. n ≥ 26 for each group. **p < 0.05. Scale bar length is 10 μm.

It has been shown that the Hedgehog pathway transcriptionally promotes *yki* expression in the follicle cell lineage^22^. Since we have demonstrated that the Yki level is increased in *ptc* mutant cells, it is important to investigate whether the Hedgehog pathway regulates nuclear localization of Yki through a post-translational mechanism. *FRT42D* control and *ptc* mutant follicle cell clones were generated and the immunofluorescent staining intensities of Yki in the nucleus or in the whole cell were quantified (Fig. 6). The ratio of nuclear Yki immunofluorescent intensity to the total intensity was significantly higher in *ptc* mutant cells than that of the *FRT42D* control clones (Fig. 6C). This result suggested that the Hedgehog pathway might regulate nuclear localization of Yki in addition to the transcriptional regulation proposed by a previous study^22^.

**Figure 7:**
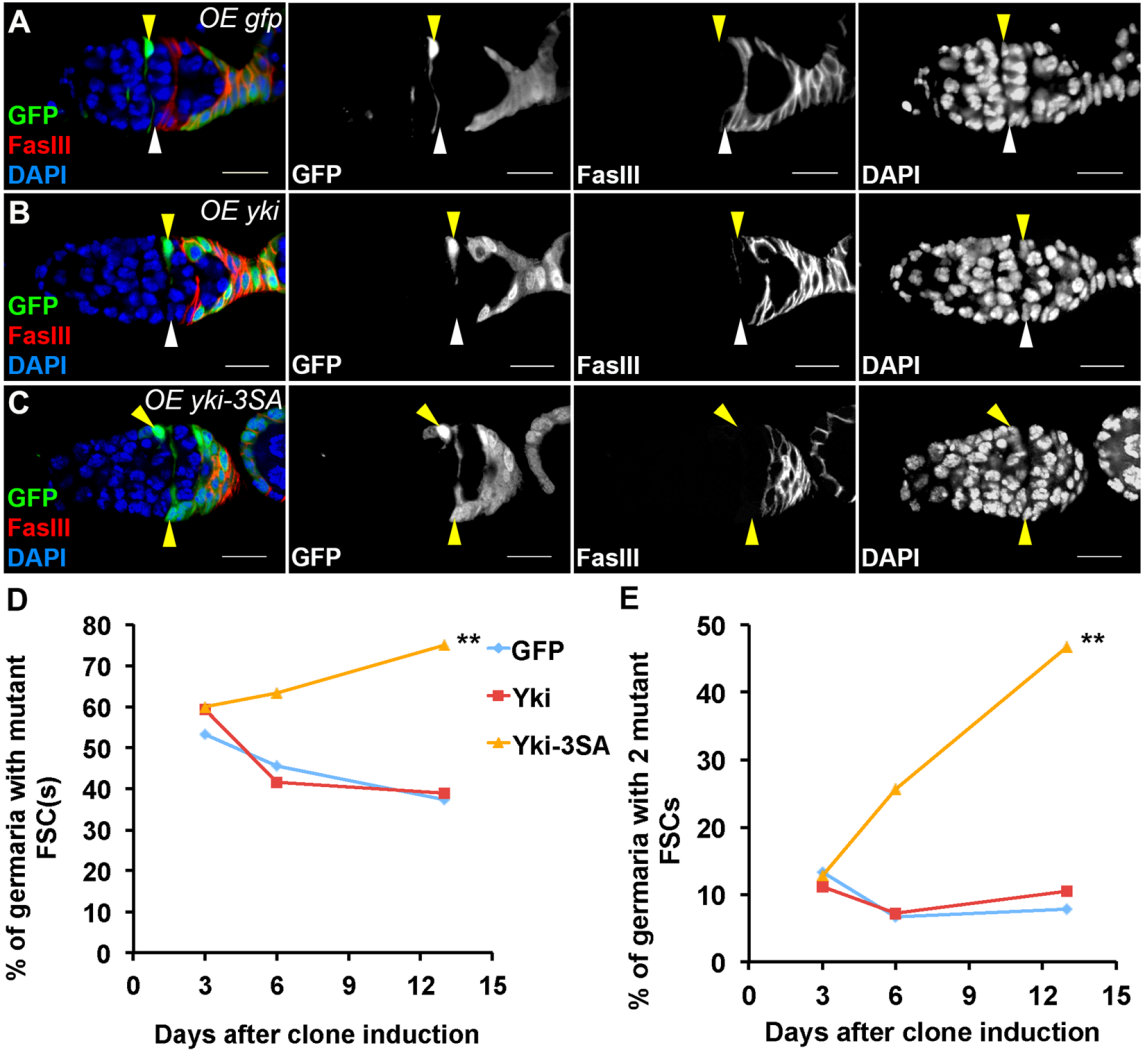
Yki phosphorylation is key for regulating FSC maintenance. Ovaries were dissected thirteen days after clone induction and stained with anti-GFP, anti-FasIII, and DAPI. FLP-out clones were GFP-positive. (**A**) A germarium with one GFP-negative FSC (white arrowheads) and one GFP-positive FSC (yellow arrowheads) in the control group were shown. **(B**) A germarium with one GFP-negative (white arrowheads) FSC and one GFP-positive FSC over-expressing *yki* (yellow arrowheads) were shown. (**C**) A germarium with two GFP-positive FSCs over-expressing *yki-3SA* (yellow arrowheads) were shown. Scale bar length is 10 μm. (**D**,**E**) The percentages of germaria with one or two GFP-positive FSCs (**D**), or two GFP-positive FSCs (**E**) over-expressing *gfp* (control), *yki*, or *yki-3SA* at different time points were shown. The percentage of *yki-3SA* group with one or two GFP-positive FSCs was significantly higher than that of *gfp* control and *yki* groups thirteen days after clone induction. Pearson’s chi-squared test was used for statistic analysis. *n* ≥ 180 for each genotype/time point.

If the Hedgehog pathway exclusively regulates FSC maintenance by increasing transcription of *yki* ^22^, increase of wild-type *yki* expression alone should be sufficient to promote FSC maintenance as *ptc* mutation does. We used the FLP-out technique with *act-GAL4* to generate clones over-expressing *yki or yki-3SA* and examine FSC maintenance. Both *UAS-yki* and *UAS-yki-3SA* were inserted into the same locus through site-specific integration to minimize the variation of transgene expression^49^. FLP-out clones expressing *gfp* were generated as a control. In the control group (*OE gfp*), most GFP-positive FSCs were maintained thirteen days after clone induction (Fig. 7A,D and E). Over-expression of *yki* showed similar FSC maintenance as the control (Fig. 7B,D and E), suggesting that increase of *yki* expression alone does not promote FSC maintenance. On the other hand, over-expression of *yki-3SA* led to a higher percentage of germaria containing one or two GFP-positive FSCs than that of the control or *yki* over-expression (Fig. 7C–E). Together with data in Fig. 6, our results suggested that increase of Yki nuclear localization, but not merely *yki* expression, promotes FSC maintenance. While activation of the Hedgehog pathway strongly promotes FSC maintenance, it is likely that the Hedgehog pathway regulates Yki through a post-translational mechanism in addition to the previously proposed transcriptional regulation^22^.

## Discussion

In this study, we show that inhibition of Yki function results in loss of the FSC and disruption of the follicular epithelium. Over-activation of Yki by either expression of a constitutively active form of *yki* or mutation of genes encoding for negative regulators of Yki such as *hpo*, *wts*, or *sav* promotes FSC maintenance. These data suggest that proper activity of the Hippo pathway is critical for FSC maintenance. However, underlying mechanisms of the Hippo pathway in regulating FSC maintenance remains to be identified. We have previously shown that inactivation of Yki promotes polar cell fate and activation of Yki disrupts polar cell differentiation^39^. Thus, it is possible that inactivation of Yki leads to premature polar cell differentiation of FSCs, therefore results in FSC loss. In addition, the Hippo pathway has been shown to regulate multiple aspects of follicle cell functions through varieties of signaling pathways. In border cells, the Hippo pathway regulates polarized distribution of the actin cytoskeleton^40, 50^. In polar cells, the Hippo pathway regulates *upd* expression to control border cell induction and migration^40^. In addition, Wts regulates invasion of follicle cells into egg chambers in coordination with basolateral junctional components, such as Fasciclin 2 and Discs large 1^51^. Thus, it remains possible that the Hippo pathway may control cytoskeleton, JAK/STAT signaling, or cellular junctions, which in turns affects FSC maintenance.

In addition to its function in the FSC, the Hippo pathway plays important roles in various types of stem cells in *Drosophila*, such as neural stem cells (NSCs) and intestinal stem cells (ISCs)^52^. At early larvae stage, NSCs may either stay in quiescence or proliferate in response to metabolic changes. Recent studies have shown that the Hippo pathway regulates the activity of several factors involved in asymmetric cell division, such as Mushroom body defect (Mud), Canoe/Afadin, and Bazooka, therefore control neural stem cell division and differentiation^53, 54^. In the intestine, the Hippo pathway is required in both niche cells and ISCs for regulating ISC proliferation, especially during injury-induced regeneration^44, 45, 55^. In either niche cells or ISCs, the Hippo pathway controls expression of *upd* to promote proliferation and survival of ISCs. These studies in other stem cells may provide hints to understand how the Hippo pathway controls FSC maintenance.

Here we show that the Hedgehog pathway acts upstream of the Hippo pathway in regulating FSC maintenance. Interestingly, although both the Hippo and Hedgehog pathways control cyst stem cell maintenance in the *Drosophila* testis, these two pathways do not interact with each other^56^. In the wing disc, mutation of *ptc* leads to overproliferation of surrounding cells through activation of Yki^57^, demonstrating that the Yki also acts downstream of the Hedgehog pathway in a non-autonomous manner. A recent study has shown that the Hedgehog controls Yki through Mastermind (Mam) at transcription level to regulate FSC maintenance^22^. They used *Diap*-*LacZ* as a reporter for Yki activity. However, the expression of *Diap-LacZ* does not reflect the activity of the Hippo pathway in the follicle cell lineage during early oogenesis (Supplementary Fig. S1A–C, Fig. S1A–D). Moreover, we did not observe changes of Yki level or nuclear localization in follicle cells either over-expression or mutation of *mam* as well as over-expression of a constitutive form of *ci* (Supplementary Fig. S4). It will be important to further investigate how Mam regulates FSC maintenance. The interaction between the Hippo and Hedgehog pathways has also been demonstrated in mammals. In cerebellar granule neuron precursors (CGNPs) and medullablastoma, activation of the Sonic Hedgehog pathway both induces Yap expression and promotes Yap nuclear localization^58^. Interestingly, activation of Yap increases the activity of the Sonic Hedgehog pathway in CGNPs and cortical progenitors, suggesting that these two pathways may form a positive feedback loop to promote expansion of neural progenitors and/or accelerate tumorigenesis. The Sonic Hedgehog pathway has also been shown to act downstream of the Hippo pathway in promoting proliferation and inhibiting neuronal differentiation in mouse cortical progenitors^59^. A previous study has demonstrated that the Hedgehog pathway regulates Yki at transcriptional level in the *Drosophila* ovary^22^. Since we observe an increase of Yki nuclear distribution in *ptc* mutant cells and over-expression of Yki alone does not promote FSC maintenance, we conclude that the Hedgehog pathway regulates Yki through a post-translational mechanism. While overall level of Yki is increased in *ptc* mutant clones (Figs 4A and 6B), we do not exclude the possibility that the Hedgehog pathway regulates Yki at transcriptional level. Thus, it is important to further investigate how these two pathways interact with each other in regulating stem cell properties.

Various upstream signals have been demonstrated to regulate the Hippo pathway, including the apical-basal polarity complex. Interestingly, we found that the level of aPKC is increased and the apical distribution of aPKC is disrupted in *ptc* mutant clones (Supplementary Fig. S5A–C), where the level and distribution of Yki are increased (Figs 4A and 6B). Therefore, it is possible that the Hedgehog pathway regulates the Hippo pathway through affecting apical-basal polarity. However, we did not observe significant change of the Yki level or nuclear distribution in aPKC knockdown follicle cells (Supplementary Fig. S5D). Since there is limitation in quantification of subtle change in Yki level or distribution based on immunofluorescence signals, it remains possible that the Hedgehog pathway regulates FSC maintenance through cell polarity. It is recently suggested that the apical-basal polarity of FSCs is unique by its broadly distributed adherens junctions and the lack of a mature apical domain. Mutation of two basolateral junction genes, *lethal giant larvae* (*lgl*) or *discs large* (*dlg*), promotes FSC maintenance^60^. While our data have shown interaction between the Hippo and Hedgehog pathways, as well as the Hedgehog pathway and cell polarity, it will be interesting to further examine how the Hippo pathway, the Hedgehog pathway, and cell polarity regulates stem cell maintenance in concert.

## Methods

### Fly stock

Fly lines used for over-expression and knockdown experiments: *P{UAS-yki.V5.O}attP2* (Bloomington stock center BLM28819), *P{UAS-yki.S111A.S168A.S250A.V5}attP2* (BLM28817), *UAS-GFP*, *P{KK109756}VIE-260B* (Vienna Drosophila Resource Center, V104523), *P{GawB}NP1624/CyO, P{UAS-lacZ.UW14}UW14* (DGRC Kyoto Stock Center, DGRC 104055), *P{GAL4}C587. tub-Gal80* ^*ts*^ */CyO* (gift from Dr. G.J Liaw). Fly lines used for clonal analysis: *hs-flp, ubiGFP FRT19A/(FM7c)*, *hs-flp*; *ubiGFP FRT40A/CyO*, *hs-flp;FRT42D ubiGFP/CyO*, *hs-flp;;FRT82B ubiGFP*, *FRT19A*, *FRT40A, ey-flp;FRT42D*, *ey-flp;;FRT82B/TM3*, *Sb*, *FRT42D yki* ^*B5*^,*w* ^*+*^ */CyO*, *w* ^*−*^; *FRT42D hpo* ^42–47^,*w* ^*+*^ */CyO, FRT82B wts* ^*x1*^ */TM3*,*Sb*, *FRT42D ptc* ^*IIw*^ */CyO, smo* ^3^ *FRT40A/CyO*, *FRT82B sav* ^3^ */TM6b*, *sd* ^*∆B*^ *FRT19A/FM7*, *w* ^*^; *P{UAS-mam.A}2*, *y* ^*d2*^ *w* ^*1118*^ *P{ey-FLP.N}2 P{GMR-lacZ.C(38.1)}TPN1*; *P{neoFRT}42D P{GT1}mam* ^*BG02477*^ */CyO y* ^*+*^, *hs-flp;;P{GAL4-Act5C(FRT.CD2).P}/TM3*, *hs-flp;UAST-GFP act > CD2 > Gal4/(CyO)*. *lacZ* reporter and other lines include *w* ^*1118*^, *y* ^*1*^ *sc* ^*^ *v* ^*1*^; *P{TRiP.HMS01320}attP2* (BLM 34332), w*; *P{lacW}ex* ^*697*^ */CyO*; *TM2/TM6B, Tb* ^1^, *y* ^*1*^ *w* ^*^; *P{lacW}Diap1* ^*j5C8*^ */TM3, Sb* ^1^ (BLM 12093).

### Antibodies and Reagents

The following antibodies were used at the indicated dilutions: mouse anti-Fasciclin III 1:200 [7G10, Developmental Studies Hybridoma Bank (DSHB)], mouse anti-β-Galactosidase 1:200 (40-1A, DSHB), rabbit anti-GFP 1:1000 (Invitrogen, USA), rat anti-GFP (1:1000, Nacalai Tesque, Japan), Dylight-488 goat anti-rabbit IgG(H + L), Dylight-549 goat anti-mouse IgG(H + L) (Jackson ImmunoResearch Laboratories, USA), Alexa-546 Phalloidin 1:50 (Invitrogen). Yki rabbit polyclonal antibody was generated with full-length Yki^29^. Rabbit anti-Cas (1:5,000) was generous gifts from Dr. Chang and Dr. Jang^48^.

### Overexpression and RNAi knockdown with UAS-GAL4 system

Flies were cultured at 18 °C before eclosion. Newly eclosed adult females were collected and incubated at 29 °C for six days before dissection.

### Generation of mitotic clones

Mitotic clones were generated by using the FLP/FRT and Flip-out systems^59, 60^. Two days after eclosion, female flies were collected and heatshocked for four times in two days. They were heatshocked twice for 30 min each with a four-hour interval on the first day. On the second day, they were heatshocked once for 30 min and once for one hour with a four-hour interval. Flies with mutant clones were then incubated at 25 °C; flies with Flip-out clones were incubated at 29 °C.

### Immunohistochemistry

Ovaries were dissected in phosphate-buffered saline (PBS) and fixed in 4% paraformaldehyde (PFA) in PBS for 15 minutes. After fixation, ovaries were washed with PBT (1XPBS, and 0.2% Triton X-100) for 3 times, followed by incubation in blocking solution PBTB (1XPBS, 0.5% Triton X-100, 5% goat serum, 2.5 mg/ml BSA, and 0.05% Sodium azide) for 1 hour. Ovaries were then incubated with the primary antibodies overnight at 4 °C and followed by secondary antibody staining for 2 hours at room temperature. Ovaries were further stained with DAPI (1 μg/ml, Sigma) in PBT for 30 minutes prior mounting with mounting solution [85% glycerol, 1XPBS, 3% propyl gallate (Sigma), and ProLong® Gold Antifade reagent (Invitrogen, Carlsbad, CA, USA)].

### Fluorescence microscopy

All the images were taken by Zeiss LSM700 confocal microscope (Carl Zeiss AG) and processed with Adobe Photoshop CS5 (Adobe). For quantification of fluorescence intensity of Yki immunofluorescence, cells with large area of DAPI staining were selected. The boundary of a cell and the nucleus were determined by FasIII immunostaining and DAPI staining, respectively. The read of average intensity in the field was measured by using ImageJ (NIH, Bethesda, MD).

## Electronic supplementary material

Supplementary Fugures
