# Supplementary material for: The Hippo pathway acts downstream of the Hedgehog signaling to regulate follicle stem cell maintenance in the Drosophila ovary
Source: Sci Rep. 2017 Jun 30;7:4480. doi: 10.1038/s41598-017-04052-6 (PMC5493701; doi:10.1038/s41598-017-04052-6)
Supplement: Supplementary file 1 — Supplementary Fugures [file 41598_2017_4052_MOESM1_ESM.pdf]

The Hippo pathway acts downstream of the Hedgehog signaling to regulate follicle stem cell maintenance in the Drosophila ovary

Ta-Hsing Hsu, Chia-Yu Yang, Tsung-Han Yeh, Yi-Chia Huang, Tsu-Wei Wang, Jenn-Yah Yu

Supplementary Figures

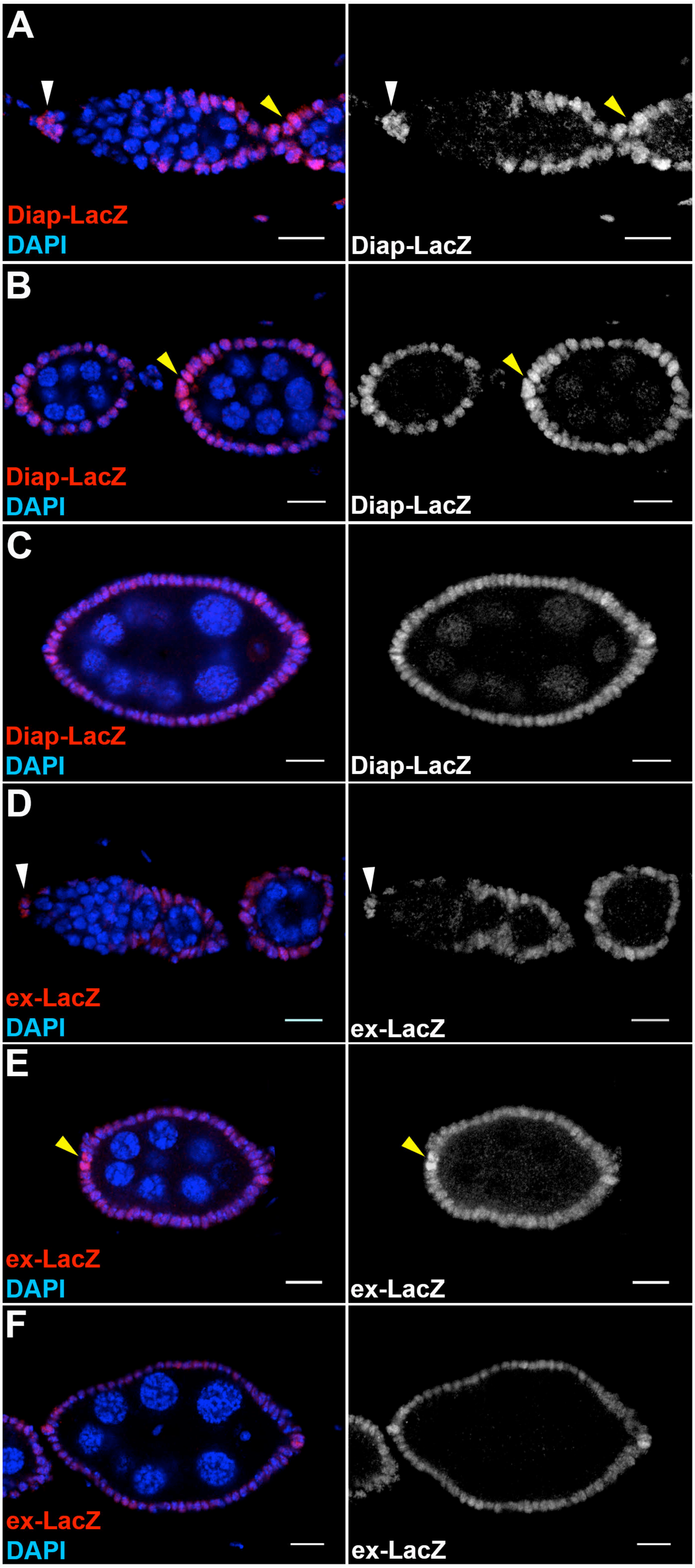
A
Diap-LacZ
DAPI
Diap-LacZ
B
Diap-LacZ
DAPI
Diap-LacZ
C
Diap-LacZ
DAPI
Diap-LacZ
D
ex-LacZ
DAPI
ex-LacZ
E
ex-LacZ
DAPI
ex-LacZ
F
ex-LacZ
DAPI
ex-LacZ

Figure S1. *Diap-LacZ* and *ex-LacZ* are expressed in cells withdrawn from mitosis of the follicle cell lineage.

Ovaries from *ex-LacZ and Diap-LacZ* were stained with anti-β-GAL in red and DAPI. All egg chambers were oriented as anterior to the left. (A-C) In *Diap-LacZ*, β-GAL was detected in cap cells at the anterior tip of the germarium (a white arrowhead in A), polar cells in egg chambers (yellow arrowheads in A and B) and cells withdrawn from mitosis at stage 7 (C). (D-F) In *ex-LacZ*, β-GAL was detected in cap cells at the anterior tip of the germarium (a white arrowhead in D), polar cells in egg chambers (a yellow arrowhead in E) and cells withdrawn from mitosis at stage 7 (F). Scale bar length is 10 μm.

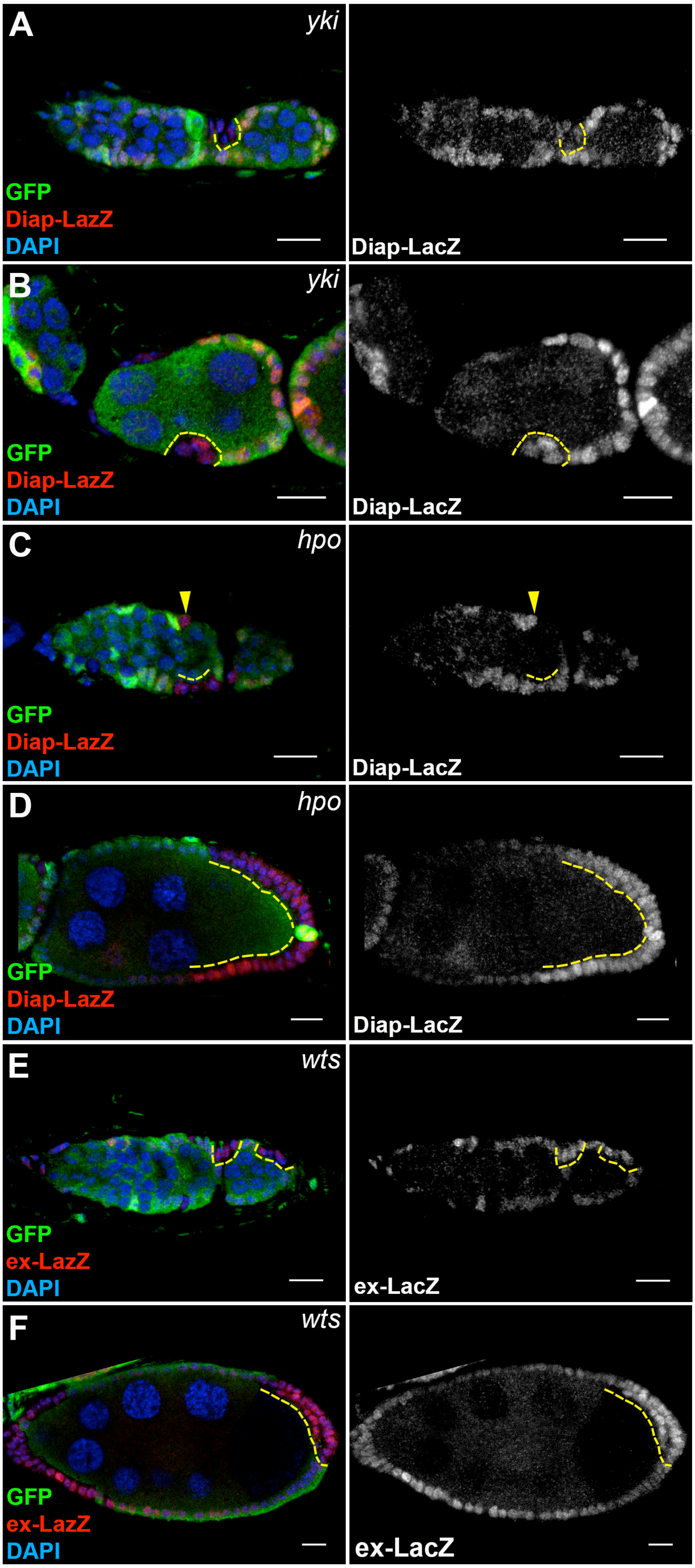
A
yki
GFP
Diap-LazZ
DAPI
Diap-LacZ
B
yki
GFP
Diap-LazZ
DAPI
Diap-LacZ
C
hpo
GFP
Diap-LazZ
DAPI
Diap-LacZ
D
hpo
GFP
Diap-LazZ
DAPI
Diap-LacZ
E
wts
GFP
ex-LazZ
DAPI
ex-LacZ
F
wts
GFP
ex-LazZ
DAPI
ex-LacZ

Figure S2. The expression of *Diap-LacZ* or *ex-LacZ* is not regulated by the Hippo pathway in the follicle cell lineage during early oogenesis.

Ovaries were dissected six days after clone induction and stained with anti-GFP, anti-β-GAL in red, and DAPI. Mitotic clones were GFP-negative. All egg chambers were oriented as anterior to the left. (A, B) *yki* mutant clones were generated in the background of *Diap-LacZ*. The intensity of β-GAL was slightly reduced in follicle cell precursors (yellow dashed line in A) and follicle cells at stage 5 (yellow dashed lines in B) in comparison with the neighboring GFP-positive control cells. (C, D) *hpo* mutant clones were generated in the background of *Diap-LacZ*. (C) The intensity of β-GAL in *hpo* mutant follicle cell precursors (yellow arrowheads and dashed lines) was similar to that of the neighboring GFP-positive control cells. (D) In the posterior egg chambers at stage 8, the intensity of β-GAL in *hpo* mutant cells (yellow dashed lines) was higher than that of the neighboring GFP-positive control cells. (E, F) *wts* mutant clones were generated in the background of *ex-LacZ*. (E) The intensity of β-GAL in *wts* mutant follicle cell precursors (yellow dashed lines) was similar to that of the neighboring GFP-positive control cells. (F) In the posterior egg chambers at stage 8, the intensity of β-GAL in *wts* mutant cells (yellow dashed lines) was higher than that of the neighboring GFP-positive control cells. Scale bar length is 10 μm.

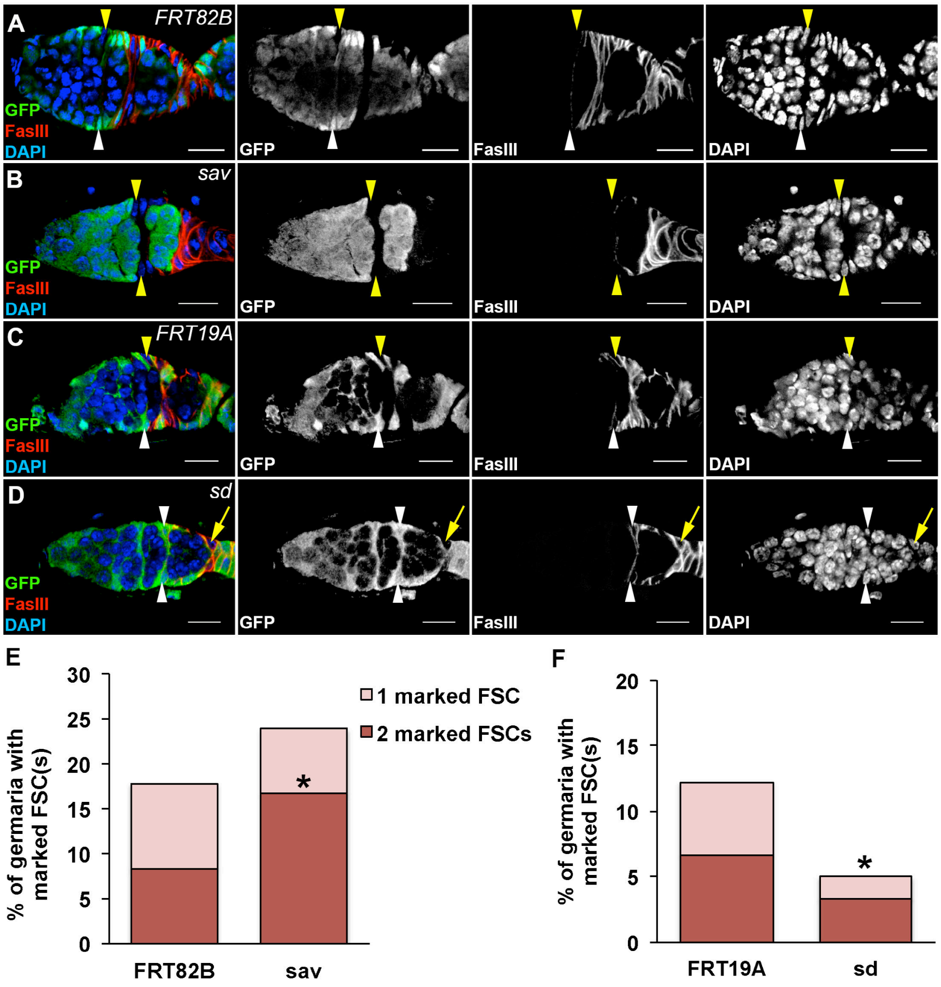

Figure S3. Sav and Sd regulate FSC maintenance.

Ovaries were dissected thirteen days after clone induction and stained with anti-GFP, anti-FasIII, and DAPI. Mitotic clones were GFP-negative. (A) A germarium with one GFP-negative (yellow arrowheads) and one GFP-positive (white arrowheads) FSCs in *FRT82B* control were shown. (B) A germarium with two GFP-negative *sav* mutant FSCs (yellow arrowheads) were shown. (C) A germarium with one GFP-negative (yellow arrowheads) and one GFP-positive (white arrowheads) FSCs in *FRT19A* control were shown. (D) A germarium with two GFP-positive FSCs (white arrowhead). *sd* mutant cells have left the niche (yellow arrows). (E, F) Germaria were categorized based on the numbers of GFP-negative FSCs in each germarium thirteen days after clone induction. The percentage of germaria with two *sav* mutant FSCs was higher than that of the *FRT82B* control. Pearson's chi-squared test was used for statistic analysis. $n \geq 180$ for each point. *: $p < 0.05$. (E) The percentages of germaria with one and two GFP-negative *FRT82B* or *sav* mutant FSCs were shown. The percentages of germaria with two *sav* mutant FSCs were higher than that of the *FRT82B* control. (F) The percentages of germaria with one and two GFP-negative *FRT19A* or *sd* mutant FSCs were shown. The percentages of germaria with one or two *sd* mutant FSCs were lower than that of the *FRT19A* control. Scale bar length is 10 μm.

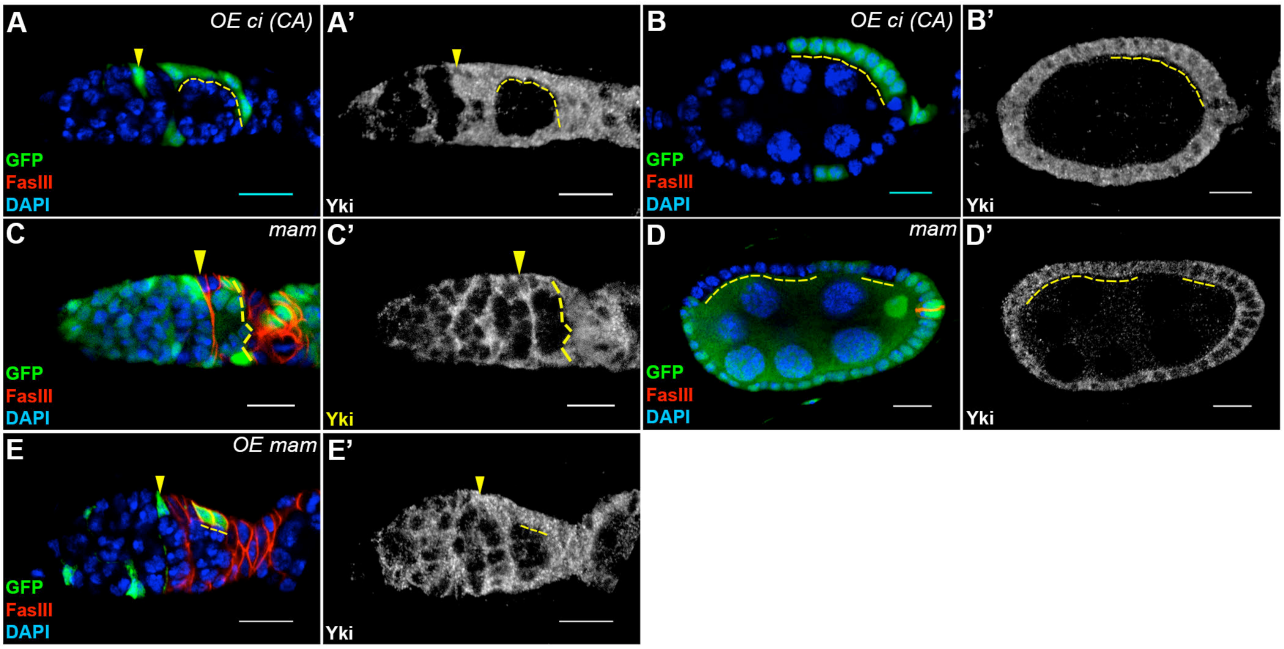

Figure S4. The Hedgehog pathway does not regulate Yki through Ci and Mam.

Ovaries were dissected six days after clone induction and stained with anti-GFP, anti-FasIII, anti-Yki, and DAPI. FLP-out clones were GFP-positive (A, B, E). Mitotic clones were GFP-negative (C, D). (A, B) The intensity of Yki immunofluorescent staining was not affected in GFP-positive cells expressing a constitutive mutant form of *ci* (yellow arrowheads and dashed lines) comparing with GFP-negative neighboring control cells in the germarium (A) or at stage 5 (B). (C, D) The intensity of Yki immunofluorescent staining was not affected in GFP-negative *mam* mutant cells (yellow arrowheads and dashed lines) comparing with GFP-positive neighboring control cells in the germarium (C) or at stage 6 (D). (E) The intensity of Yki immunofluorescent staining was not affected in GFP-positive cells expressing *mam* (yellow arrowheads and dashed lines) comparing with GFP-negative neighboring control cells in the germarium. Scale bar length is 10 µm.

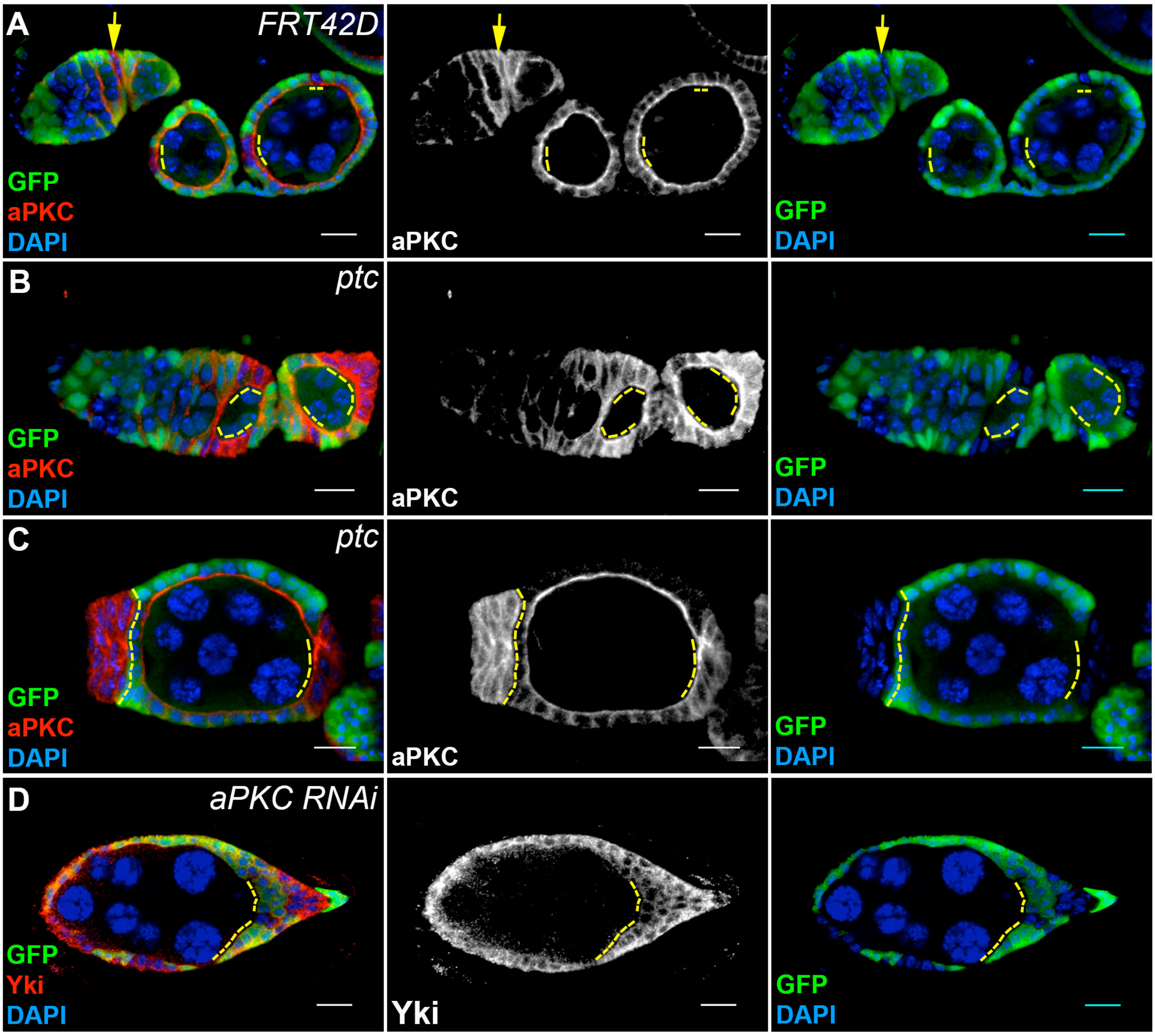

Figure S5. The Hedgehog pathway does not regulate Yki through modulating the apical-basal polarity.

Ovaries were dissected six days after clone induction and stained with anti-GFP, anti-aPKC (A-C), anti-Yki (D), and DAPI. Mitotic clones were GFP-negative (A-C). FLP-out clones were GFP-positive (D). (A) The pattern of aPKC immunofluorescent staining in GFP-negative *FRT42D* control cells in the germarium and stage 2/4 egg chambers (yellow arrows and dashed lines). (B, C) The intensity of aPKC immunofluorescent staining was increased in GFP-negative *ptc* mutant cells (yellow dashed lines) comparing with GFP-positive neighboring control cells in the germarium, stages 2 and 5 egg chambers. (D) The intensity and distribution of Yki immunofluorescent staining was not affected in GFP-positive *ptc* knocked down cells (yellow dashed lines) comparing with neighboring GFP-negative control cells at stage 6. Scale bar length is 10 µm.
